# Tuberculosis and Slight Ailments

**Published:** 1899-11-04

**Authors:** 


### Tuberculosis and Slight Ailments.

Among the many points which have of late been rendered manifest by the divers discussions which have taken place on the subject of tuberculosis, none is more important than the fact that many of the slight ailments from which people of all conditions suffer from time to time are really attacks of tuberculosis from which recovery takes place. This is, of course, no new discovery. The recognition in the post-mortem room of the dried-up results of bygone attacks of tuberculosis, if not as old as the hills, is at least as old as most of our knowledge of this disease. What is important, and what is being insisted upon now more than it used to be, is the fact that the deposition of these nodules, the occurrence of these attacks of localised tuberculosis, is the cause of slight ailments taking the form of debility, anæmia, or what not, which, after a time, may pass away without having shown any symptoms directly pointing to the lungs as the seat of the disorder.

It has always been recognised that long before the occurrence of those definite symptoms and physical signs which used to be demanded before one felt justified in "condemning" the patient as being affected with consumption, a condition of debility often complicated with dyspepsia had usually existed for a considerable time. In fact, such a condition was spoken of by some as a distinct "pre-tubercular stage" of the malady, and was rightly regarded as the stage in which one could most hopefully count upon a cure. There is, however, every reason to believe that the "pre-tubercular" stage of the older authors really represents the stage in which the initial infection of tuberculosis is taking place, and that many of the symptoms which used to be accepted as indicative of actual tuberculosis are rather the result of the implantation of other infections in and around the area which has already been gradually invaded by the tubercle bacillus. Professor Allbutt put the matter very clearly in a recent discussion. "For long enough," he says, "perhaps for months, the proper tuberculous process may be plugged in an enclosed area without access to the outer world." In the sputum of such cases there may be no tubercle bacilli. As these bacilli develop slowly in the affected area, the tissues resist the disease, the mass, the "knot," resulting from this resistance may become more densely encapsulated, may dry up and calcify, and the whole disease, so far as symptoms are concerned, may entirely pass away, without ever having been recognised as having had to do with pulmonary tuberculosis. As Allbutt says, "A person may be a little 'off' colour' for a few weeks or months; or he is 'bilious,' 'anæmic,' or 'over-worked'; he coughs a little, or not at all, no tubercle is found in the sputum, even if sought, and he recovers by encapsulation, unaware that the shadow of the black hawk's wing had rested upon him."

Perhaps it is true, it probably is, that anæmia and debility and other blood conditions predispose to phthisis, but it must not be forgotten that the anæmia or debility, by the curing of which we often seem to save a patient from consumption, is in no small number of cases the result of tubercle already deposited, and that what has appeared to be a recovery from anæmia has in reality been a recovery from tuberculosis. It may be said, how can this be proved? Perhaps it cannot be proved, but the frequency with which scarred lungs are found in persons whose history tells no tale of serious disease is alone sufficient to show how comparatively slight must be the symptoms which sometimes accompany the occurrence of an attack of tuberculosis which, although localised, has been very marked.

All this brings us round to the importance of careful examination and serious treatment even of slight ailments. Of the cases that get well, never to be discovered until they are brought to light by a necropsy years and years afterwards, how many would not have gone on to phthisis if they had been exposed to the causes which commonly lead to the breakdown of tuberculous masses, such as deficient feeding, overwork, and especially exposure to bad air—air charged with animal exhalations, and carrying into the lungs with every breath those secondary infections which, when implanted upon a tuberculosis, tend to turn it into a consumption? Modern teaching about the treatment by fresh air, by ample feeding, and by the air of mountains, contains much that is hopeful to those actually suffering from consumption; but, if this teaching is to have its full effect in saving life, it must be applied to tuberculosis before consumption has come on, and for this purpose we must always be on the look-out for early cases, by careful observation of temperature and by thorough examination of the most vulnerable portions of the lungs, even in slight ailments which at first sight may seem to show no evidence of having any relation to so serious a disease. And when in such cases it is found that there are signs and symptoms which suggest an early stage—which, be it observed, often means a curable stage—of tuberculosis, what are we to do? It is a difficult position. It is so easy to let things slide; it seems so simple a matter to patch up a case like this with tonics and a little change of air; it seems so hard to tear a young fellow from his employment just when he is getting his foot on the ladder; and the proverbial ingratitude of patients makes it so dangerous a matter to label as tuberculous a patient who may in six months' time be perfectly well, when he assuredly will hold one up to ridicule for making so much of a case of "mere debility and overwork." And added to all this there is the doubt, for at such a stage the symptoms can never be very certain. Yet, if good is to be done, now is the time, and the only time, at which any certain prospect of cure can be held out. Under such circumstances, we must "grasp the nettle." We have no right to assume that amid the conditions in which the disease has developed it will get well.

